# *Euglena*, a Gravitactic Flagellate of Multiple Usages

**DOI:** 10.3390/life12101522

**Published:** 2022-09-29

**Authors:** Donat-P. Häder, Ruth Hemmersbach

**Affiliations:** 1 Department of Botany, Emeritus from Friedrich-Alexander University, 91096 Erlangen, Germany; 2 German Aerospace Center, Institute of Aerospace Medicine, Gravitational Biology, Linder Hoehe, 51147 Cologne, Germany

**Keywords:** *Euglena*, flagellate, graviperception, gravitaxis, gravitational biology, regenerative life support system, in situ resource utilisation, lunar habitat, Mars exploration

## Abstract

Human exploration of space and other celestial bodies bears a multitude of challenges. The Earth-bound supply of material and food is restricted, and in situ resource utilisation (ISRU) is a prerequisite. Excellent candidates for delivering several services are unicellular algae, such as the space-approved flagellate *Euglena gracilis*. This review summarizes the main characteristics of this unicellular organism. *Euglena* has been exposed on various platforms that alter the impact of gravity to analyse its corresponding gravity-dependent physiological and molecular genetic responses. The sensory transduction chain of gravitaxis in *E. gracilis* has been identified. The molecular gravi-(mechano-)receptors are mechanosensory calcium channels (TRP channels). The inward gated calcium binds specifically to one of several calmodulins (CaM.2), which, in turn, activates an adenylyl cyclase. This enzyme uses ATP to produce cAMP, which induces protein kinase A, followed by the phosphorylation of a motor protein in the flagellum, initiating a course correction, and, finally, resulting in gravitaxis. During long space missions, a considerable amount of food, oxygen, and water has to be carried, and the exhaled carbon dioxide has to be removed. In this context, *E. gracilis* is an excellent candidate for biological life support systems, since it produces oxygen by photosynthesis, takes up carbon dioxide, and is even edible. Various species and mutants of *Euglena* are utilized as a producer of commercial food items, as well as a source of medicines, as it produces a number of vitamins, contains numerous trace elements, and synthesizes dietary proteins, lipids, and the reserve molecule paramylon. *Euglena* has anti-inflammatory, -oxidant, and -obesity properties.

## 1. Introduction

### Characteristics of the Genus Euglena

**Figure 1:**
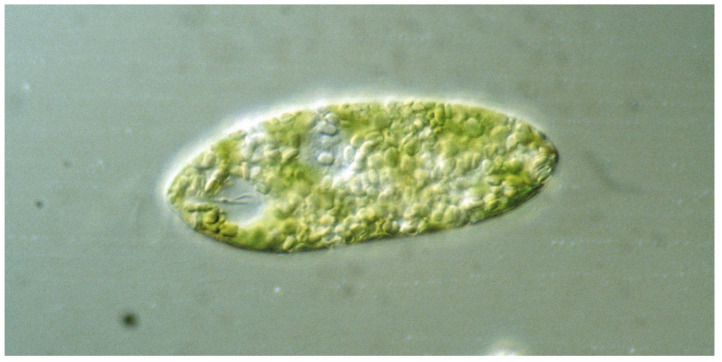
*Euglena gracilis*, as seen under a transmission light microscope (Zeiss Axioplan, 40× objective) showing the nucleus, chloroplasts, reservoir with the two flagellar bases, and the paraflagellar body (PFB). One flagellum is seen outside the reservoir. The cell is 80 µm long.

The genus *Euglena* contains unicellular eukaryotic flagellates with more than 50 genera and over 800 species in the class Euglenoidea dwelling in marine and freshwater habitats [1,2,3]. The species are robust and tolerant to variable environments, climate change, and low temperatures. Under adverse conditions, such as dryness, neustonic *Euglena* species can form permanent cysts, called palmella stage [4]. The cells have an elongated ovoid form of about 100 µm length. Most *Euglena* species have two flagella originating in the basal bodies at the bottom of an indention at front end, called the reservoir (Figure 1). In most species, only one flagellum exits to power forward movement in a trailing manner, while the other is very short and ends inside the indention. The flagellum caries 10,000 hair-like filaments, called mastigonemes [5]. In *E. mutabilis* and some other species, both flagella do not leave the reservoir, so that these forms are restricted to gliding motility [6,7]. In, e.g., *E. gracilis,* near the basis of the emerging flagellum, a prominent red spot can be seen, which consists of carotenoids granules. While initially this “eyespot” was thought to be responsible for light direction detection, its nature as photoreceptor has been ruled out (see below) [8]. Under optimal conditions, *Euglena* species can form dense blooms, such as the green *E. gracilis, E. viridis, E. pascheri*, or *E. tuba* [9,10], as well as the red colored *E. sanguinea* [11]. The latter has been cultivated in raceway ponds on the basis of an organic medium enriched with mineral fertilizers to produce biodiesel [12].

The genus looks back to a billion years-long evolutionary history, including significant horizontal gene transfer, which facilitates a complex metabolism and cell biology. The original forms were probably heterotrophic unicellular organisms, which later on obtained the ability of photosynthesis. The widely accepted “chloroplast symbiont hypothesis” claims that heterotrophic eukaryotic organisms incorporated cyanobacteria-like photosynthetic prokaryotes, which were the precursors of chloroplasts [13]. Indications for this hypothesis are the presence of cyanobacteria-like DNA, which is responsible for the synthesis of some of the chloroplast proteins and bacterial ribosomes, as well as a double membrane. The outer membrane is contributed by the eukaryotic host, and the inner one represents the cyanobacterial membrane. In contrast, the photosynthetic *Euglena* species contain chloroplasts with a triple membrane, which indicates that the heterotrophic phagotrophic ancestors may have acquired their chloroplast via a secondary symbiosis ingesting a eukaryotic partner [14]. The chloroplasts contain chlorophylls *a* and *b*, as well as pyrenoids, which store paramylon. In darkness, *Euglena* can grow heterotrophically in the presence of organic material. Some species or mutants have lost their chloroplasts, either naturally or induced artificially, and are restricted to a phagotrophic life [15]. Sexuality has never been observed in *Euglena*, so that reproduction is limited to asexual cell division: first the nucleus divides; then, the cell splits lengthwise into two daughter cells starting at the front end. During this process, the stigma disappears; later, two new ones appear, which are distributed to the daughter cells. [16]. The emergent flagellum shortens until it is no longer visible, and the resulting daughter cells regenerate two flagella each.

Many phytoplankton orient themselves, with respect to environmental clues, to optimize their position in the water column, either by active motility and steering or passive movement changing their buoyancy [17]. *E. gracilis* has the capacity to use stimuli from the environment for orientation. Orientation, with respect to light, is an obvious advantage for photosynthetic organisms. The stigma is not the photoreceptor, but the paraflagellar body (PFB) located at the basis of the emerging flagellum [18]. During forward locomotion in lateral light, the stigma casts a periodic shadow onto the PFB, since the cells move in a helical fashion. This modulated light signal is used to trigger an angular course correction, until the cell’s long axis is aligned with the light direction [19]. At low light intensities, the cells move toward the light source (positive phototaxis); at high intensities, they switch to negative phototaxis (away from the light source) [20]. After decade-long discussions regarding the nature of the photoreceptor in *E. gracilis* phototaxis, Iseki and Watanabe identified the pigment as a blue-light-activated adenylyl cyclase responsible for the photophobic response, resulting in photoaccumulation or photoavoidance [21]. Later on, it was shown that these molecules are also responsible for phototaxis [22,23]. The complex signal transduction chain for phototaxis in *E. gracilis* has been detailed in a recent review [19]. The cells also respond to oxygen (aerotaxis) and carbon dioxide gradients (chemotaxis) [24]. These responses may explain an earlier observation, where *E. gracilis* cells accumulated in a red light field [25]. The red light may have resulted in photosynthetic oxygen production, by which the cells were attracted from outside the light field. The cells also orient themselves perpendicular to magnetic field lines and move toward a high field in a magnetic gradient (magnetotaxis) [26]. Finally, *E. gracilis* displays a pronounced gravitaxis (see below) [27,28]. In order to understand the complex behavior to environmental stimuli, it is important to analyze the interaction of the various responses to light, gravity, and chemical stimuli.

The human exploration of space and other celestial bodies involves many challenges that have to be solved. Harsh and restricted living conditions in space vehicles or habitats demand technical requirements to maintain human health and provide nutrient supply. Earth-bound supply of material and food is restricted, and in-situ resource utilisation is a prerequisite. Excellent candidates for playing variable roles and delivering several items are unicellular algae, such as the space-approved flagellate *E. gracilis*.

## 2. Graviperception and Graviresponses in *E. gracilis*

### Physiology of Gravitaxis

During evolution, all organisms were exposed to the gravitational field of the Earth and, consequently, have developed mechanisms to sense and respond to the direction of gravity [29,30,31,32]. Early observations showed that motile microorganism, though heavier than water, are capable of swimming upward in a gravitational field [33,34], a behavior termed negative gravitaxis because it guides the cells away from the center of gravity. A number of hypotheses have been established to explain the phenomenon [35,36,37,38,39], including the so-called buoy effect: the cells were thought to be tail-heavy, so that the flagellum would pull the cell upward [40,41,42]. However, a number of observations casted doubt on this hypothesis: microscopic analysis did not show any asymmetry in the cell body, young cells in their logarithmic growth phase shortly after inoculation move downward (positive gravitaxis) [28], and, furthermore, negative gravitaxis can be inverted by the presence of heavy metal ions, such as copper, mercury, cadmium, or lead [43,44,45]. In contrast, exposure of older cells to excessive visible radiation or UV reverses negative gravitaxis into a positive one [46,47]. This sign change in gravitactic orientation is not mediated by the photoreceptor, since it was also found in cells that lack the pfb (colorless and blind mutants), but it is brought about by reactive oxygen species (ROS, probably hydrogen peroxide), as revealed by using the fluorescent probe 2′,7′-dichlorodihydrofluorescein diacetate. Flushing the cells with nitrogen, which removes the oxygen, or the application of Trolox, potassium cyanide, or ascorbic acid, which scavenge ROS, suppressed sign change of gravitational orientation [48]. Extended exposure to solar radiation decreases the precision of gravitaxis [46,49]. Even though *E. gracilis* is adapted to freshwater, it tolerates salinity of up to 19 g/L. During this treatment, the swimming speed and precision of gravitaxis decreases. At salt concentrations above 15 g/L, negative gravitaxis changed to a positive one. However, it is interesting to note that the cells kept showing positive gravitaxis, even after transfer back into freshwater medium [50].

**Figure 2:**
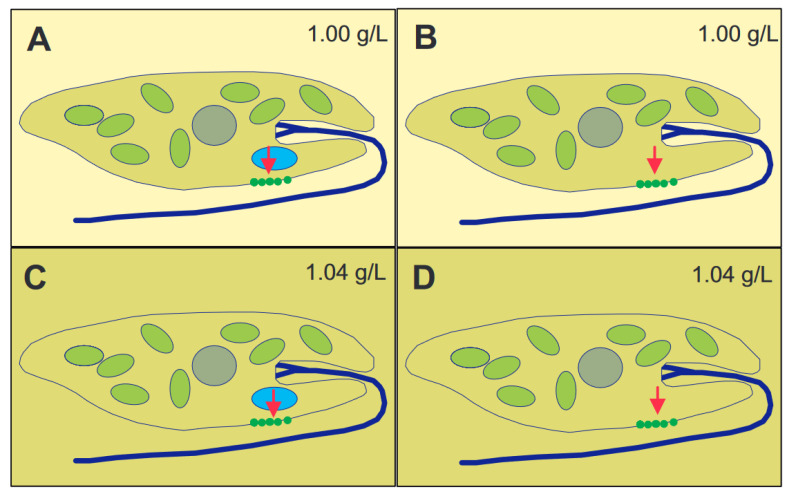
Principles of graviperception, either by means of a distinct gravisensor (here statolith) or the whole cell mass as sedimenting parameter. A statolith (blue) presses (red arrow) onto mechano-sensitive ion channels (**A**,**C**) or the whole cell content exerts pressure (red arrow) onto the channels (**B**,**D**). When the cells, having a specific weight of 1.04 g/L, are in a lower-density medium (1.00 g/L) (**A**,**B**), the pressure will open the channels, and the direction of gravi-stimulus will be detected. When the cells are under isodensity conditions, with respect to the surrounding medium ((**C**,**D**), 1.04 g/L), only the pressure of the statolith will be detected, but not that of the whole cell content.

The long axis of immobilized cells killed by liquid nitrogen pointed in random directions [51]. These, and other, results indicate that gravioriention in *E. gracilis* is based on an active perceptive mechanism. Some organisms use heavy bodies inside the cell with operate as statoliths [52,53]. Since no obvious sedimenting bodies could be found in *E. gracilis* by microscopic analysis, the alternative is that the whole cytoplasmic content of the cell with its organelles acts as a statolith [54,55]. The specific weight of the cells was determined by isopygnic centrifugation in tubes with layers of increasing Ficoll concentrations [56]. The specific density of the cells was found to be between 1.045 and 1.054 g/mL, depending on the culture age and conditions, as older cells were heavier than those in newly inoculated cultures. There is an elegant experiment to distinguish between the action of a heavy statolith within the cell or the whole cytoplasm (Figure 2) [57,58]. In case of a statolith, which presses onto a sensor inside the cell, it does not matter if the cell is in a 1.00 g/mL medium or Ficoll at 1.04 g/mL. In contrast, if the cell with a specific weight of 1.04 g/mL floats in a medium of the same density, it will not perceive the gravity pull, while it does so in 1.00 g/mL medium. Since *E. gracilis* showed a response according to the latter scheme, it was clear that the whole cytoplasmic content pressed onto the lower membrane. Cells starved for over 600 days had a specific density of 1.011 g/mL and did not display any graviorientation [59].

Like many other organisms, *E. gracilis* shows a dominant circadian rhythm, which is expressed in many physiological, biochemical, and behavioral processes [60,61]. Under constant light and temperature conditions, individual cells are not synchronized, and no circadian rhythm was detected; however, when the cells were exposed to a circadian light/dark change, the precision of gravitaxis followed the rhythm with a minimum in the darkness and maximum in the early afternoon [62,63]. The fact that the precision of orientation increased, even before the light was switched on, indicates that the internal circadian rhythm was entrained by the light/dark change [64]. In addition, the form of the cells (elongated vs. rounded), swimming velocity, and internal concentration of cyclic adenosine monophosphate (cAMP) followed the circadian rhythm (see below for the role of cAMP in graviperception). *E. gracilis* cells can also be synchronized to much shorter light/dark cycles, down to 1:1 h [65].

## 3. Gravireceptor and Molecular Sensory Transduction Chain in *E. gracilis*

### 3.1. Mechanosensitive Channels and Calcium

The important question aims at the characterization of the molecular nature of the gravireceptor. Mechanosensitive ion channels belong to a large family of transient receptor potential proteins (TRP), which are found in numerous organisms, from bacteria to humans, having different functions, such as photoperception, nociperception (pain sensation), taste, thermosensation, fluid flow detection, tactive sensation, and mechanosensing [66,67]. The TRP channel proteins have six membrane spanning alpha helices with an ion-specific pore [68]. In order to identify the involvement of a specific TRP channel in graviperception, RNA interference (RNAi) was applied [69]. The technique is based on the fact that a short, double-stranded RNA (19−23 nucleotides) causes a post-transcriptional gene silencing, which is sequence-specific. Since this RNA sequence binds to the specific mRNA of the targeted protein in an antisense manner, it blocks its function and inhibits the synthesis of the corresponding protein. Using this technique, involving degenerative primers aiming at the pore region of the mechanosensitive channels, four transcripts in *E. gracilis* were obtained. After cloning, sequencing and BLAST analysis, it turned out that three of the four transcripts did not represent motifs of TRP channels, while one had a similarity to the C-terminal end of a TRP channel [68]. Using RNAi for the first three PCR products did not disturb the gravitaxis in *E. gracilis*, while the fourth one completely impaired graviorientation; this inhibition lasted for about one month. Mechanosensitive ion channels are opened by minute mechanical forces and allow the transport of ions through the cell membrane. The pressure of the cellular content onto the TRP channels in the membrane is sufficient to open them and allow the influx of specific ions. Assuming a specific cell density of ~1.05 g/mL, the force *F* can be calculated using the equation

(1) $$F=V\times g_{n}\times \Delta \rho$$

where *V* is the volume of the cell, *g_n_* the acceleration, and Δ*ρ* the specific density difference between the cell body and medium [70]. Using the measured values for *E. gracilis* indicates that the cell content presses onto the mechanosensitive channels in the lower membrane, with a force of 0.57–1.13 pN [56]. It should be stressed that, during forward locomotion, the cell rotates around its long axis at about 1 rpm, which results in a pulsed signal, as long as the cell deviates from the vertical orientation.

Earlier results had shown that *E. gracilis* uses a calcium-specific ATPase to pump Ca^2+^ out of the cell. The resulting internal concentration of 10^−9^ M is about 1 million times lower than that of the surrounding water [71]. Inhibition of this pump using vanadate impairs gravitaxis because the Ca^2+^ gradient across the membrane decreases, and opening of the mechanosensitive channels allows only a limited Ca^2+^ influx [57,58,72]. Blocking the TRP channel with gadolinium also reduces the precision of gravitactic orientation [73]. Likewise, the incorporation of calcimycin (A23187), an artificial calcium ionophore, into the cell membrane inhibits gravitaxis, since it breaks down the Ca^2+^ gradient [27,58]. Additionally, in *Chlamydomonas*, mechanosensitive Ca^2+^ channels have been found [74], and a mutant that lacks these channels did not show gravitaxis [75]. If a gated Ca^2+^ influx is, indeed, involved in gravitactic reorientation, there should be a transient increase in the intracellular calcium concentration. This can be visualized by a fluorescent chromophore (Calcium Crimson) after loading it into the cells by electroporation [76]. Since, in the green *E. gracilis*, the intensive chlorophyll fluorescence could mask the calcium signal, the colorless, but gravitactic competent *E. longa* and several colorless and gravitactic *E. gracilis* mutants were chosen [64]. After the cells had been adapted within the cuvette, it was inverted, and the cells started to reorient, accompanied by a strong fluorescent signal near the reservoir. Spectrofluorometric monitoring confirmed an increase in the Calcium Crimson fluorescent signal [77]. These results were supported during parabolic airplane maneuvers [78]. At 1 g, the cells showed an intermediate calcium signal, which increased after the transition to 1.8 g and decreased during microgravity. A gravity-dependent calcium signal was also found on a 13 min lasting MAXUS 3 sounding rocket parabolic flight [79], where the cells were in microgravity conditions that were experimentally interrupted by defined centrifugal accelerations of 0.1, 0.2, or 0.3 g, respectively. As expected, the application of the blocker gadolinium strongly reduced the calcium signal, since it impairs mechanosensitive ion channels [80]. The position of the TRP channels has not yet been pinpointed, but theoretical considerations indicate that they must be located at the front end of the cell, underneath the trailing flagellum, since a course correction is initiated when the flagellum points downwards.

During gated calcium influx, the membrane potential of the cell should decrease (more positive ions inside). So far, no one has yet been able to measure the inside cellular potential of *Euglena* with an electrode. Alternatively, one can follow the electrochromic absorbance bandshift of Oxonol VI loaded into the cells, which changes its absorption between 590 and 610 nm during membrane potential changes [81]. A photometer was built using sets of LEDs emitting these two wavelengths, and the signal was determined with phototransistors [82]. Indeed, the cytoplasm was found to become more positive during gravistimulation, which is an indication that, during reorientation of the cells (monitored by computer-controlled cell tracking), positive charges (Ca^2+^) enter the cells [83]. Application of EGTA sequesters calcium ions in the outer medium. This resulted in reduced gravitaxis and diminished membrane potential change, as measured during a parabolic flight campaign [84]. Finally, the micromolar application of the lipophilic cation triphenylmethyl phosphonium inhibited gravitaxis [84], since it passes the membrane and reduces the internal negative potential [85].

A gene for a mechanosensitive channel in *Saccharomyces* has been sequenced [86]. Various primers were used against a gene extracted from *E. gracilis* using PCR, and four sequences were obtained resembling the *Saccharomyces* gene [87]. In total, over 1500 PCR products were isolated, which were cloned in plasmids and sequenced [68]. While most PCR products coded for proteins with other functions, one was found that corresponds to the TRP channel and is a good candidate for the mechanosensitive ion channel operating as gravisensor in *E. gracilis*.

### 3.2. Calmodulin

Calcium controls many biochemical reactions in most living organisms, including bacteria, plants, and animals [88]. Calcium ions can bind to calmodulins, proteins of about 150 amino acids [89,90], usually with four calcium binding sites [91]. The binding site motifs comprise 12 amino acids forming a loop (EF motif) [92]. A total of five different calmodulin genes were found [93], one of which had already been characterized in *E. gracilis;* this calmodulin is located under the cell wall, called pellicula, where it could be involved in euglenoid gliding motility and controlling the cell form [94]. By sequencing the genes of all five calcium binding proteins (CaM.1–CaM.5), it was found that all were different, but all contained the typical EF motif. The dsRNA of each gene was inserted in different *E. gracilis* samples by electroporation, which yielded different results. RNAi using CaM.1 caused strong cell form abnormalities, and the cells could not swim, but only crawl [68], even though they had a visible flagellum. RNAi with CaM.2 had a similar effect, but it was restricted to only a few days, after which the cells returned to normal swimming motility. However, as they did not show gravitaxis; this calmodulin is thought to be responsible for the gravitactic signal transduction. RNAi with CaM.3–CaM.5 did not yield an obvious effect on the cell morphology and motility of gravitactic orientation [93]. Immunoblotting and indirect immunofluorescence using an anti-CaM.2 antibody revealed that CaM.2 is located both in the cell and flagellum of *E. gracilis* [95]. Calmodulin can be specifically inhibited by trifluoperazine or fluphenazine, as well as W7 [96,97,98]. Application of these substances also inhibited gravitaxis in *E. gracilis* [75]. It is interesting to note that a calmodulin was also found in the flagellum of *Chlamydomonas* [99].

### 3.3. Cyclic Adenosine Monophosphate

As indicated above, the cellular cAMP concentration changes in parallel to the precision of gravitaxis during the circadian rhythm in synchronized *E. gracilis* cultures [62]. Its involvement in the signal transduction of gravitaxis was shown in a dedicated sounding rocked space experiment on TEXUS 36 [100]. The 1 mL cell suspension was filled into 2 mL syringes, which were connected by a tube to another 2 mL syringe containing 1 mL ethanol as a fixative, but separated by a rubber ball. A total of 112 assemblies were mounted on a centrifuge, providing three different acceleration (g) levels for some periods during the 7 min-lasting microgravity phase of the rocket flight. At predefined times and different g levels, the fixative was injected into the cell suspension in groups of 8–12 syringes in parallel by hydraulic pressure. The intracellular cAMP concentration was analyzed after the retrieval of the TEXUS payload using a radioimmunoassay on a scintillation counter. The cAMP concentration was low in microgravity at accelerations up to 0.08 g. This value had been found to be the threshold for gravitactic orientation in *E. gracilis* during the IML-2 mission on the American shuttle Columbia. The cAMP concentration increased significantly at higher acceleration of 0.12 and 0.16 g, respectively [100,101]. In addition to the controls, several *E. gracilis* syringes contained 1 mM gadolinium chloride, an inhibitor of mechanosensitive calcium channels [57,102]. In these samples, no increase in cAMP was stimulated by centrifugation, even above the threshold >0.12× g. In another set of samples, 10 mM caffeine was added, a known inhibitor of phosphodiesterases [58,103,104]. This treatment resulted in a tripling of the cAMP concentration in microgravity, but no further increase could be detected during gravitactic stimulation, indicating that the responsible adenylyl cyclase could probably not provide a further increase in concentration. Indomethacine, a specific inhibitor of the adenylyl cyclase [105], decreased the precision of gravitaxis in *E. gracilis* [75,106]. In contrast, forskolin activated the adenylyl cyclase [107] and increased the precision of gravitactic orientation in *E. gracilis* [75]. It is interesting to note that cAMP is also involved in gravitactic signal processing in the ciliate *Paramecium* [108,109], as well as in controlling motility and development of slime molds [110,111]. Phosphodiesterase quenches the cAMP, so that the cellular concentration decreases when the *E. gracilis* cells are aligned with the gravity vector. The enzyme can be impaired by caffeine, theophylline, or IBM-X, which results in an increase in cellular cAMP [112,113]. Therefore, the application of these substances augments gravitaxis [114]. The phosphodiesterase cannot degrade 8-bromo-cAMP, which is an artificial analog and substitute of cAMP, so that also this drug increases the precision of gravitaxis in *E. gracilis* [58]. The photoreceptor for phototaxis, the light-induced adenylyl cyclase, also produces cAMP. This could indicate that the two sensory transduction chains, for phototaxis and gravitaxis, converge at this point, and the following steps, including the control of the flagellar movement, are identical.

### 3.4. Protein Kinase A and Course Correction

Thus, the next question is how cAMP induces a change in flagellar motility to induce a change in swimming direction. Favaro et al. found that cAMP induces a protein kinase A (PKA) [115]. Protein kinases can be inhibited by stauropsin [116], which, in fact, abolishes negative gravitaxis in *E. gracilis* [117]. However, about 3 to 4 h later, the cells show positive gravitaxis [118]. Stauropsin also inhibits phototaxis, another indication that the steps in the sensory transduction chain for phototaxis and gravitaxis, starting with cAMP, are obviously identical.

Using degenerative primers showed that *E. gracilis* produces at least five different versions of protein kinase A dubbed PK.1–PK.5, which were sequenced using RACE-PCR [118]. Specific dsRNA was produced against each of the five genes and inserted into cells by electroporation, in order to perform RNAi. Application of RNAi against PK.4 inhibited gravitaxis for three weeks, after which, positive gravitaxis ensued. As expected, it impaired also phototaxis. In contrast, RNAi against the other isoforms had no effect. The protein kinase A is supposed to phosphorylate a motor protein in the flagellum, which results in a change in the beating pattern [119].

There are about 1700 flagellar proteins in *E. gracilis* [120]. A specific protein, EgPCDUF4201, has been identified to be involved in gravitaxis, since silencing either the C- or the N-terminus by RNAi inhibited the orientation of the cell [95]. It is interesting to note that this protein also interacts with CaM.2. Immunoblotting and indirect immunofluorescence using a genomic PKA antibody showed a localization in the lower part of the flagellum inside the reservoir [121].

**Figure 3:**
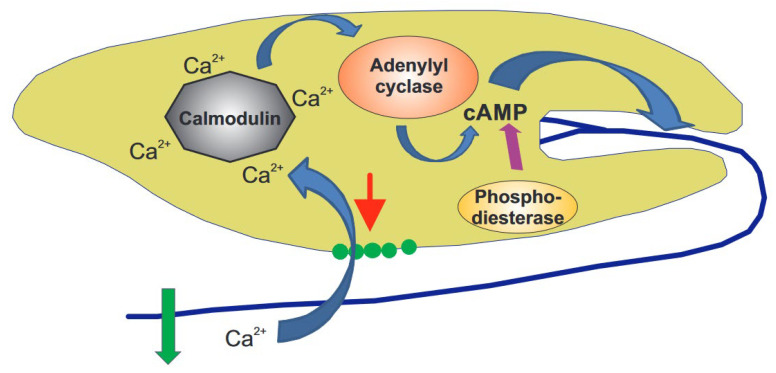
Putative sensory transduction chain of gravitaxis in *E. gracilis*. When the cell content presses (red arrow) onto the mechano-sensitive channels (green dots), while the flagellum points downwards during rotation, they open and allow a gated Ca^2+^ influx. This calcium binds to a calmodulin, which, in turn, activates an adenylyl cyclase. This enzyme converts ATP to cAMP, which activates a protein kinase A, which is thought to activate proteins in the flagellum and swings out (green arrow) to induce the course correction before a phosphodiesterase breaks down the cAMP (purple arrow) to quench the stimulus.

The observations detailed above can be combined into a complex gravitaxis signal transduction chain in this flagellate (Figure 3) [122]. Since the cell content has a higher specific density than the outer medium, it applies pressure onto mechanosensitive TRP channels, which are assumed to be located at the front end, underneath the trailing flagellum, as long as the cell’s long axis deviates from the vertical. Since the cell rotates around its long axis, a modulated signal occurs whenever the flagellum points downwards. The resulting gated calcium influx binds to a specific calmodulin (CaM.2), thereby activating an adenylyl cyclase that produces cAMP from ATP. Shortly after activation, cAMP is broken down by a phosphodiesterase. This transient spike in cAMP controls a specific protein kinase A, located in the flagellum, responsible for the change in movement and course correction. The sensory transduction chains for phototaxis and gravitaxis in *E. gracilis* seem to converge at the production of cAMP. The involvement of the individual components has been proven by RNAi, as well as specific inhibitors.

## 4. Methods to Modify the Influence of Gravity

Various tools and platforms have been constructed to alter the influence of gravity and study its impact on biological systems. New concepts and hypotheses can, thus, be approached, thereby paving the way for long-term experimentation in microgravity, in order to unravel the impact of gravity on life on Earth and other planets.

### 4.1. Hypergravity as a Tool to Identify Gravity-Related Processes

Often, gravity-related processes are hard to identify. Increased gravitational stimulation can be achieved by linear acceleration using centrifugation. Centrifugation in a moderate and physiological range (up to 10 times Earth gravity) is provided by custom-made centrifugation platforms, thus allowing for observation, the cultivation of cells, plants, and small animals, and even the exposure of larger systems, such as humans or flight hardware [123]. Responses to increased gravitational stimulation, adaptation processes to altered gravity, and re-adaptation to normal gravitational conditions provide new insights into perception mechanisms and signaling pathways. Furthermore, the testing of acceleration phases during the launch and landing phases are prerequisites before performing space experiments. The impact of gravity on the motility and orientation of *E. gracilis* has been observed in the slow rotating centrifuge microscope (NIZEMI) during accelerations between 1 g and 5 g. At 1 g, the cells show a weak negative gravitaxis, which becomes more pronounced at higher accelerations, up to about 3 g. Most of the cells were capable of swimming, even against an acceleration of 4.5 g, though a passive downward movement, due to the acceleration force, started. Even higher accelerations in the range of 10 g, during, e.g., rocket launches (TEXUS), did not affect vitality of the swimming cells [124].

### 4.2. How Much Gravity Force Is Needed for a Gravi-Response?

Regarding the possibilities of experimentation in hypergravity, the questions arise—why do we need experiments in real weightlessness (0 g), and why can we not just extrapolate data from different acceleration points to zero? Thresholds characterize the physiological processes in living systems, thereby making the behavior at 0 g and, thus, stimulus-free conditions—a unique and unpredictable situation. The threshold for acceleration-related responses is one of the important parameters used to, e.g., characterize the underlying processes of sensing gravity [125]. The existence of thresholds in the range of 0.1–0.3× g of various graviresponses was proven by exposing plants, cells, and microorganisms on the centrifuge microscope NIZEMI during the IML-2 (International Microgravity Laboratory) Space Shuttle STS-65 mission [126]. Threshold studies, by means of centrifuges, are also operated on rockets (TEXUS, MAXUS), as well as in ISS modules, such as the ESA European Modular Cultivation System (EMCS) and BIOLAB [127,128].

Furthermore, centrifugation in microgravity using on-board centrifuges offers the possibility to perform 1 g reference experiments to discriminate gravity effects from radiation effects. Additionally, partial gravity can be applied to study the effects of lunar (ca. 0.16 g) or Martian (ca. 0.38 g) gravity. Experiments were performed under these partial gravity conditions during the Joint European Partial-G Parabolic Flight program by adaptation of the flight angle and overall velocity of the plane [129]. Even a complete compact satellite, EU:CROPIS (*Euglena* and Combined Regenerative Organic-Food Production in Space), provides lunar and Martian gravity conditions by adaptation of the spin for stabilizing the satellite accordingly. Although, as of today, in 2022, the satellite is still successfully flying, allowing, for the first time, a long-term radiation measurement at 600 km height, the sophisticated life support system, containing *E. gracilis* for oxygen supply, could not be activated for technical reasons [130].

The existence of thresholds for the gravitactic behavior of *E. gracilis* proved the existence of a physiological mechanism triggering a controlled vertical movement (positive or negative gravitaxis). Richter et al. determined the threshold acceleration for the gravitactic response in *E. gracilis* as being in the range between 0.08 and 0.12 g [131,132].

### 4.3. Neutralization of the Influence of Gravity

Experimentation under real microgravity conditions is unique; however, it is possible to mimic, to some extent, weightlessness conditions in an Earth-bound laboratory. Sedimentation is the fundamental gravity-related effect coupled to a sensory mechanism, such as sedimentation of the cell mass of *E. gracilis* that triggers signaling pathways via mechanosensitive ion channels, second messengers, and finally, the gravity-determining response, in the case of *E. gracilis* “gravitaxis”. As a consequence, an environment that prevents the sedimentation and registration of the unidirectional gravitational stimulus will simulate the situation as it occurs in real microgravity. Of course, every applied method should not induce non-gravitational side effects, which themselves might trigger responses and bear the risk of misinterpretation of the results.

As an example, magnetic levitation, as a simulation approach for microgravity studies on biological (cellular) samples, failed, as proven by exposing *E. gracilis*. Online microscopical observation of swimming and immobilized *E. gracilis* in vertical high magnetic fields was expected to show that the clearly visible negative gravitaxis converts into random swimming, as known from studies in real microgravity conditions. Furthermore, immobilized cells should no longer sediment in an appropriate simulation condition [133]; however, this was not the case during magnetic levitation. Due to the fact that living objects contain diamagnetic and paramagnetic substances, the inhomogeneous magnetic field might induce strange sensations during levitation of the object, which is not the case in weightlessness. A direct comparison of the chlorophyll-containing *E. gracilis* versus chloroplast-free species, such as *Astasia longa (=E. longa)*, revealed that the persisting passive orientation in the magnet was determined by the structure with the highest anisotropy—in this case, obviously the chloroplasts [133].

Rotation-based devices are another approach to prevent sedimentation or randomize the influence of gravity, assuming that the system does not perceive the rotating gravity vector as a stimulus. To what degree an object really experiences “weightlessness” depends on the sensitivity of its gravity perception mechanism. Clinostats, random positioning machines (RPM), and rotating wall vessels (RWV) are devices for gravitational biology research in the preparation of space experiments and have been reviewed in detail [134,135,136,137].

The differences and characteristics of these different devices are in the number of rotation axes and applied operational mode. The 2D clinostats rotate samples around one axis, perpendicular to the direction of the Earthly gravity vector. Samples of preferentially low size are located along the axis of rotation to keep centrifugal accelerations as small as possible [135,138]. The speed has to be adjusted to prevent sedimentation, as well as centrifugal forces. The portfolio of the 2D clinostat types is broad enabling, e.g., the cultivation of cells in suspension or adherent cells, online measurement of kinetic responses, or online live cell imaging during exposure.

Addition of a second axis of rotation describes a 3D clinostat or random positioning machine (RPM) depending on the operation mode. A 3D clinostat, per definition, rotates with a constant speed and direction. Random speeds, with random directions of the two axes, define a random positioning machine (RPM) [139,140]. Application of the bioluminescent dinoflagellate *Pyrocystis noctiluca*, as an indicator for mechanical stress, identified a significantly higher stress response on an RPM, compared to its behavior on a fast-rotating 2D clinostat [141,142]. These results indicate the limitations of simulations if induced shear stress at the membrane surface is likely to be an important variable affecting how the cells sense gravity [143,144]. A further rotation-based simulation platform is the rotating wall vessel, characterized by a relatively large tube of about 20 cm diameter, continuously rotating perpendicular to the Earthly gravity vector, to warrant that samples passively follow the rotation without sedimenting or being centrifuged [145].

*E. gracilis* has been exposed on fast rotating 2D and 3D clinostats equipped with microscopical observation. Both approaches revealed the loss of negative gravitaxis and, thus, the loss of orientation, comparable to the behavior in real microgravity. However, the increased and persisting mean swimming velocities, especially during 3D clinorotation indicate a mechanostimulation of this highly sensitive cell type under the chosen simulation restrictions [146,147].

This short overview of simulation methods demonstrates the necessity to verify results from ground-based simulation approaches by ones obtained in real microgravity conditions to avoid misinterpretations and choose the appropriate simulation approach for each test system. Control experiments, with respect to shear stress, are necessary to exclude non-gravitational side effects. In any case, the alteration of the constant influence of gravity increases our knowledge on gravity-dependent physiology and allows for the optimal preparation of space experiments [134,135,140].

## 5. Real Microgravity

The gold standard in gravitational biology is to perform experiments in real weightlessness. Some responses to environmental stimuli can even be seen more clearly under microgravity and have led to the discovery of previously unrecognized mechanisms. However, as this situation can hardly be achieved, real “microgravity (µg)” is the used nomenclature, as the residual accelerations cannot be excluded. However, the residual acceleration should remain below the detection threshold, in order to induce physiological responses, due to the lack of gravity, sedimentation, and convection. In humans, different physiological targets have been identified, thus resulting in health issues, such as bone loss, muscle atrophy, or effects linked to altered fluid distributions in the body, raising the question about the gravity impact on single cells. Unicellular model systems, such as gravitactic protists, e.g., *E. gracilis*, clearly demonstrate the impact of the loss of gravity on cellular behavior and orientation, as well as the alterations of distinct signaling pathways. In the following, we will provide a short summary on the characteristics of the platforms that provide real microgravity conditions.

Short-term microgravity of excellent quality, in the range of 10^−4^–10^−5^ g, is provided in drop towers: no acceleration before the onset of free-fall, which lasts for only a few seconds (2.1 to 10 s). One example is the drop tower ZARM (Zentrum für Angewandte Raumfahrttechnologie und Mikrogravitation, Bremen, Germany). In the case that the µg time is doubled by a catapult system, this short, but potential, impact of a maximal acceleration of about 30 g has to be considered, with respect to the potential impact on the response.

Repetitive parabolic maneuvers of aircrafts result in around 22 s lasting periods of microgravity, with a quality of about 10^−2^ g, interrupted by periods of 1.8 and 1 g. *E gracilis* cells were exposed to this acceleration profile during parabolic flights, and their movement and physiological parameters were studied, revealing changes in the beating pattern, as well as the fast and precise adaptation of the swimming behavior. Gravity-dependent changes in the intracellular calcium concentration, characterized by a pronounced calcium influx during reorientation of the cells, identified this step as a primary event in the gravity sensing of *E. gracilis* [78,148]. Ongoing studies revealed that even the gene expression in *E. gracilis* alters under parabolic flight conditions [149]. Already, the ~20 s acceleration phase resulted in around two-fold up- or down-regulations of particular genes in *E. gracilis*. Different phases of the parabolic flight affected the different gene groups involved in signal transduction, calcium signaling, stress-response, membrane, and cytoskeletal proteins.

Sounding rocket flights in Europe are dominated by the German and Swedish programs, including TEXUS (Technologische Experimente unter Schwerelosigkeit), Mapheus (Materialphysikalische Experimente unter Schwerelosigkeit), Mini-TEXUS, MASER (Materials Science Experiment Rocket), and MAXUS (long duration sounding rocket programme) campaigns, launched from the ESRANGE launch site near Kiruna, Sweden.

Depending on the type of rocket and apogee of the flight, microgravity times from 3–4 min (Mini-TEXUS), 6–7 min (TEXUS, MASER, MAPHEUS), or 13 min (MAXUS) are achieved. The ascent phase of a TEXUS or MAPHEUS rocket lasts for about 70 s, with a maximum linear and spin acceleration of about 9 g before the free-fall period starts, which is terminated by the re-entry, with an acceleration of about 26 g. The prolonged experimental times in microgravity allow for a broad spectrum of physiological and molecular experiments, thereby significantly contributing to our current knowledge in cellular gravity sensing by exposing *E. gracilis* to this environmental condition [108]. It is necessary to mention that a possible influence of hypergravity and vibration during launch and landing has to be tested individually by ground-based studies, in order to avoid misinterpretation of the results.

Long-term biological studies in microgravity were possible by the development of automatic satellites, platforms (e.g., Shenzhou) and human-tended space laboratories (e.g., space shuttles, Spacelab, Spacehab, MIR, and ISS). For 15 years, MIR orbited at a height of around 400 km, with a speed of 28.000 km/h, which was followed by the International Space Station (ISS) in the same orbit. Supply vehicles, external platforms, and specific racks offer experimental conditions for dedicated and systematic studies in microgravity. *E. gracilis* cells were cultivated in the Simbox incubator on the Shenzhou 8 spacecraft in November 2011. At dedicated time points, cells were chemically fixed. Transcription data revealed several genes involved in signal transduction, oxidative stress, cell cycle regulation, and heat shock responses [63].

This short overview demonstrates that various experimental platforms and technologies are available, thus providing various times of microgravity, in order to answer scientific questions.

## 6. Biological Life Support Systems

### 6.1. Terrestrial Models

Human exploration of space and journeys to other celestial bodies pose many challenges, which have to be solved. Harsh and restricted living conditions in space vehicles or habitats demand technical requirements to maintain human health and provide nutrient supply. The Earth-bound supply of material and food is restricted, and in situ resource utilisation is a prerequisite.

When astronauts fly to Mars or other distant celestial bodies, they need to carry food, large amounts of oxygen [150], and the means to remove the exhaled carbon dioxide [151], while water is being recycled, as already performed on long-term space stations [152]. One way of solving part of the problem is employing photosynthetic microorganisms, which utilize solar radiation to produce oxygen and simultaneously take up excess carbon dioxide. Häder and Kreuzberg [153] proposed using an algal bioreactor. This concept was based on the supply of external CO_2_ and fresh media. Chemical and physical parameters, such as temperature, oxygen concentration, and temperature were monitored by electrodes. In addition, the motility and gravitactic orientation of the *E. gracilis* cells was determined by computerized tracking.

The subsequent model was completely closed, with no need to add nutrients or CO*_2_* from the outside [154]. The 11-L tank allowed for long-term cultivation and was operated for more than 600 days [155]. In addition to the parameters listed above, the absorption spectra could be measured to quantify the cellular concentration of chlorophyll and carotenoids. Toward the end of the experiment, a zoological component was added with 15 snails (*Biomphalaria glabra*) and 4 fish (*Xiphophorus helleri)* as consumers, but the fish were fed automatically [156]. The *E. gracilis* and animal compartments were separated by a membrane that passed oxygen and carbon dioxide in opposite directions. During the first five months, the *E. gracilis* cell density increased and subsequently decreased to the initial value to sharply decline toward the end of the experiment. The percentage of motile cells and their swimming velocity stayed fairly constant, but the precision of gravitactic orientation gradually decreased. The probable reason for the decrease in gravitaxis was that the density of the cells steadily decreased with the time in the container. The photosynthetic oxygen production was found to be enough to keep the animals alive.

### 6.2. Bioregenerative Life Support System in Space

As a next step, a closed environmental life support system (Aquacells) was developed for space experiments on the Russian FOTON satellite M2. A *E. gracilis* suspension was housed in a 1450-mL cylindrical container, launched in May 2005 [157], and irradiated with red LEDs to sustain photosynthesis [158]. The water from the fish tank (26 larval cichlids, *Oreochromis mossambicus*) was pumped through membrane tubes spanning the *E. gracilis* aquarium for the exchange of oxygen, carbon dioxide, and ammonia excreted by the fish. The motility and orientation of both fish and algal cells were recorded at regular intervals on video tape during the mission. The hardware was installed in the FOTON satellite and launched on a Soyuz rocket from Baikonur (Russia) for an 11-day mission. As expected, in microgravity, the *E. gracilis* cells swam randomly and at higher velocities than under 1 g conditions [157]. After this prolonged time in space, the cells took several hours to again show normal gravitaxis, which is in contrast to the short TEXUS missions, where normal gravitaxis was observed immediately after the cells were returned to the ground. Under microgravity, the cells were more rounded than in the ground control. The oxygen production was sufficient to sustain the fish. The next system, OMEGAHAB (*Oreochromis Mossambicus-Euglena Gracilis*-Aquatic HABitat), was launched for a 12-day orbital flight mission on the Russian FOTON-M3 [156].

In cooperation with Chinese scientists, a closed aquatic ecosystem (60 mL) was developed for the Shenzhou 8 spacecraft. The module contained the green algae *Chlorella*, *E. gracilis*, and three snails (*Bulnius*) in separate chambers [63]. The spacecraft flew in orbit for 17.5 days, and one snail survived. During the same mission, *E. gracilis* cells were fixed 40 min after launch in microgravity with an RNA lysis buffer. In parallel, cells that had been kept on a 1 g reference centrifuge were fixed [63]. After returning the samples to ground, the transcription of genes involved in signal transduction, oxidative stress defense, cell cycle regulation, and heat shock responses were analyzed using quantitative PCR. The results showed that *E. gracilis* responded to microgravity; of the 32 tested genes in total, 18 genes were up-regulated. These results confirm that long-term space flights are valuable tools to study the behavior, physiology, and genetics of motile microorganisms, which promise further insight into the complex molecular machinery of graviperception, signal transduction, and movement control [65,159].

A more recent development is the Eu:CROPIS [130]. It contains a nitrifying trickle filter which produces fertilizer from urine using lava rock as biofilm carriers. The fertilizer is used to rear tomatoes from seeds in a miniature greenhouse. *E. gracilis* is used as a supplementary oxygen producer, especially during the germination period [130]. For further review see [160,161,162,163,164,165,166].

## 7. *Euglena* as Commercial Product and a Source for Medicine

Starting last century, microalgae have been exploited as producer of biomass for food, fiber, feed, fertilizer, and fuel [167,168], as well as extracted cellular biomolecules, because they can be cultivated on sites not suitable for agriculture, their fast biomass production, and high protein content [169]. The green alga *Chlorella* and the cyanobacterium *Spirulina* were among the first unicellular photosynthetic organisms grown by a number of different technologies, such as open ponds, raceways, and photobioreactors [170,171]. *Euglena* entered the field of commercial production in 2005, when a Japanese company, Euglena Co., Ltd. (Minato-ku, Tokyo, Japan), was established with a small productivity that was gradually scaled up, until the first food products containing *Euglena* were marketed two years later [169]. The European Union evaluated the safety of dried whole cell *E. gracilis* as a novel food pursuant (Regulation EU 2015/2283) and concluded that there is no health risk [172]. While *E. gracilis* needs vitamins B1 (thiamine) and B12 (cobalamin) for growth and proliferation, it is a source of many other human vitamins, such as the water-soluble vitamins B2, B6, C, niacin, pantothenic acid, folic acid, and biotin, as well as the fat-soluble vitamins A, D, E, and K [173,174]. Both the photosynthetic strain Z and its bleached mutants also contain numerous trace elements, such as Al, P, S, K, Ca, Fe, Cu, and Zn [175]. It was also found to produce dietary proteins, lipids, and the β-1,3-glucan paramylon [176]. The latter is found only in euglenoids and has already been marketed as an immunostimulatory agent in nutraceuticals [177]. A number or strategies have been developed to optimize *Euglena* for industrial production [178,179]. Recently, *Agrobacterium tumefaciens* was used to induce the nuclear transformations of this biotechnologically important microalga [180]. Another approach is employing transgene-free targeted mutagens and single-stranded oligodeoxynucleotide-based knock-in using CRISP-associated nuclease 9 [181,182]. *E. gracilis*, in conjunction with chlorophytes, has also been used to convert biowaste leachates to valuable biomass and lipids [183]. The “Green Bioprinting” approach embeds microalgae of the species *Chlamydomonas reinhardtii* in 3D printed alginate-based scaffolds. Under illumination, cell number, chlorophyll content, photosynthetic activity, and, thus, oxygen release increased during further cultivation. Multichannel plotting combined human cells and algae established a co-culture system, in which the algae, as an oxygen provider, are cultivated in close vicinity to human cells—a method with potential for new therapeutic and regenerative concepts [184].

In addition, *Euglena* has been identified to be a rich source of medically beneficial products. *E. gracilis* was found to stimulate *Faecalibacterium* in the gut, thereby inducing increased defecation [185]. It also has anti-inflammatory, -oxidant, and -obesity properties [186,187]. An oral administration of a partially purified water extract from *E. gracilis* was found to prevent lung carcinoma growth in mice by decreasing the myeloid-derived cell number [188,189]. Paramylon nanofibers from the WZSL mutant of *E. gracilis* had an anti-fibrotic effect on liver damage induced by CCl_4_ in mice [190]. The paramylon has also been shown to have a renoprotective effect in chronic kidney disease in a rodent [191]. Intake of *E. gracilis* also has positive effects on mood, autonomic activity, and sleep quality [192]. Finally, paramylon has been shown to accelerate wound healing in the skin when being applied as a film dressing [193].

## 8. Conclusions and Outlook

*E. gracilis* is a remarkable versatile microalga that has been analyzed in numerous studies to reveal the mechanisms of phototactic and gravitactic orientation. It was proven that these strategies to optimize its position in the water column are based on complex molecular processes forming sensory transduction chains, which ultimately converge and result in stepwise reorientation, with respect to the stimulus direction. The organisms can be used in bioregenerative life support systems for long-term space travel, since they produce oxygen and absorb carbon dioxide by their photosynthetic machinery. Terrestrial and space experiments are underway to optimize the systems for Mars missions and the development of Moon habitats. *E. gracilis* has been proven to be non-toxic for humans and provide vitamins, minerals, and paramylon, which can be used in food additives, feed, biodiesel, and fertilizer. In addition, *E. gracilis* can be grown on a large commercial scale to produce medicinal ingredients, which have been found to have anti-cancer, -inflammatory, -oxidant, and -obesity properties in rodent models. Future applications in the treatment of human diseases require substantial research and monitoring to exclude any adverse reaction.
